# #Everything Will Be Fine. Duration of Home Confinement and “All-or-Nothing” Cognitive Thinking Style as Predictors of Traumatic Distress in Young University Students on a Digital Platform During the COVID-19 Italian Lockdown

**DOI:** 10.3389/fpsyt.2020.574812

**Published:** 2020-12-15

**Authors:** Laura Giusti, Anna Salza, Silvia Mammarella, Denise Bianco, Donatella Ussorio, Massimo Casacchia, Rita Roncone

**Affiliations:** ^1^Department of Life, Health and Environmental Sciences, University of L'Aquila, L'Aquila, Italy; ^2^Hospital S. Salvatore, University Unit Rehabilitation Treatment, Early Interventions in Mental Health, Abruzzo, Italy

**Keywords:** COVID-19 outbreak, narrative psychiatry, online psychological intervention, digital platform, traumatic impact, thinking styles, university students, predictors

## Abstract

On March 10, 2020, Italy announced its lockdown caused by the novel coronavirus (COVID-19) pandemic, and home confinement exposed individuals to a stressful situation of unknown duration. Our study aimed to analyze the emotional and cognitive experiences and the psychopathological symptoms of young Italian University students seeking help from our University student Counseling and Consultation Service during the COVID-19 lockdown. Also, our study aimed to identify the predictors of traumatic psychological distress, investigating variables that could influence the students' well-being, related to their socio-demographic and clinical condition, to the “exposition” to the social distancing, and related to their cognitive thinking style. One-hundred and three University students were included in our study. The traumatic impact was assessed by the Impact of Event Scale-Revised (IES-R). A digital platform was used in our study, focused on narrative dimensions analyses. Our results showed that 21.4% of our help-seeking students experienced lockdown as a traumatic experience. The main stressful factors reported by students were: adjustment to the new academic activities (23.3 %), lack of autonomy (19.4%), and conflicts with family members (6.8%). The three main areas impaired were: changes in the sleeping pattern (68%), difficulty in concentration (67%), and loss of energy (58.6%). Furthermore, 36% of our student sample reported being suffering from anxiety symptoms, whereas 26% showed depressive symptomatology. Students having previous psychological and psychiatric contacts with mental health services (23%) showed a more severe traumatic and depressive symptomatology. The problematic thinking style “all or nothing” was predominantly associated with psychological distress, anxiety, depression, and posttraumatic symptoms. “Everything Will Be Fine” could be identified by the “optimistic style” (27.2%), inversely correlated with the psychopathological measures and concentration problems. The results of the logistic regression analysis indicated that the length of home confinement (second month) seemed to increase by over 3 times the likelihood of experience posttraumatic symptomatology, and a thinking style “all or nothing” was the final strongest predictor increasing the risk by over 5 times. The implementation of psychological interventions to improve the mental health of vulnerable young subgroups to contain the structuring of psychopathological profiles represent a fundamental challenge.

## Introduction

At the beginning of 2020, originating from Wuhan city, coronavirus disease (COVID-19) started to spread throughout China. On January 31, two Chinese tourists in Rome, Italy, tested positive for COVID-19. In the beginning, most Italians thought that the problem could be limited to a few cases and looked suspiciously at Chinese people in our country, considering COVID-19 as a “slightly more severe form of normal flu.”

On February 18, 2020, the first case of SARS-CoV-2 pneumonia was diagnosed in Italy in an Italian man at the Codogno Hospital (Lodi) (1).

Based on the Italian government ordinance “I stay at home,” since March 10 (decree issued on March 9, 2020, by the Italian Government, identifying the so-called “Phase one”), an isolation strategy was implemented to limit the spread of the virus in Italy.

The gradual easing of Italy's lockdown began on May 4, with the reopening of manufacturing activities taking around 4.4 million workers back out of their homes, and the “Phase two” of the Italian measures to contain the spread of COVID-19 began.

Italy became one of the significant COVID-19 hotspots, and, as of May 28, a total of 231,732 people tested positive for COVID-19; furthermore, 33,142 people died (including 165 physicians), out of a population of about 60 million.

Since Italy's COVID-19 lockdown, a range of containment measures was urgently adopted, including closure of all schools and Universities and home confinement. On the one hand, it was a useful strategy for defending and protecting lives; on the other hand, the resulting distress could cause significant emotional problems of still unknown duration (2).

The risks and fear of getting infected, being worried about one's family members in other areas contracting COVID-19, cognitions, and preventive behaviors regarding COVID-19 add to other psychological pressures. Being forced to stay at home; smart working; study at home for teenagers, children, and University students using remote learning methods to drastically reduce outings and social interactions; and the uncertainty of the future were all potential stressful conditions. COVID-19 is a complex emergency that requires a dynamic interpretation of the psychological impact. This emergency has led to restrictions on physical spaces, loneliness, family problems and conflicts in restricted private areas, and a sense of vulnerability and precariousness that changes the set of priorities in both family lives and the macro-system.

Two weeks after the World Health Organization announced the emergence of a new coronavirus (2019-nCoV) as a public health emergency, Liang et al. (3) reported that nearly 40.4% of their sampled youths were found to be prone to psychological problems, and 14.4% showed post-traumatic symptoms.

Women, students, and poor self-rated health status were significantly associated with a more significant psychological impact of the outbreak and higher levels of stress, anxiety, and depression; women reported significant higher post-traumatic symptoms, in the domains of re-experiencing, negative alterations in cognition or mood, and hyperarousal (4).

A recent study on the psychological impact of the COVID-19 pandemic on college students in China reported that around 25% of their sample showed anxiety with different severity levels significantly correlated with negative effects on daily life and delays in academic activities. The authors identified “living in urban areas,” “family income stability,” and “living with parents” as protective factors and “having relatives or acquaintances infected with COVID-19” as a risk factor (5).

Home confinement or quarantine reduces the availability of timely psychological intervention and implies the interruption of traditional “face-to-face” psychological counseling. Psychological emergency, in response to health emergency, requires urgent need for new types of psychological and mental problem intervention strategies potentially feasible and accessible (6).

In the last decade, the use of digital platforms and digital health interventions has increased rapidly. The growing rate of technology access highlights the potential for treatment and engagement with services to be taken from the clinic into the context of an individual's everyday life, unconstrained by location and time (7). Furthermore, researchers have been incorporating cognitive behavioral therapy (CBT) within apps and websites to help people self-manage their difficulties and provide help and psychoeducation about anxiety or depressive symptoms (8). In the public health emergency context, digital tools offering CBT intervention can also help build resilience (2).

Narrative research has significant implications for practice in recovery-oriented mental health care (9). Sharing individual stories of psychological distress has become a central practice within recovery-based healthcare, allowing to reorganize what is confusing to individuals and reinforcing empowerment and self-determination (10).

Remote written counseling by using a “structured letter therapeutic approach” as a potentially effective strategy was proposed in the actual global health emergency context (6).

An interesting model hypothesized that narrative elements integrated in CBT practices could not only help ameliorate distress but also promote resilience, happiness, courage, and other positive qualities (11).

We hypothesize that young University students could feel severely distressed about the social isolation imposed due to COVID-19, in a phase of their life in which their peer group and interpersonal relationships have a significant impact on their emotional development and in establishing intimate relationships. Our study aimed to analyze the emotional and cognitive experiences and the psychopathological symptoms of young Italian University students seeking help, during the COVID-19 lockdown. Also, our study aimed to identify the predictors of traumatic psychological distress, investigating variables that could influence the students' well-being, related to their socio-demographic and clinical condition, to the “exposition” to the social distancing, and to their cognitive thinking style.

## Materials and Methods

### Study Design

This study was conducted through the digital platform of the Counseling and Consultation Service for Students, SACS, of the University of L'Aquila (Italy) (12).

Located in Central Italy in the town administrative center of the Abruzzo Region, the University of L'Aquila is a public teaching and research institution offering a full range of academic programs including biotechnologies, sciences, economics, engineering, education, humanities, medicine, psychology, and sport sciences. With seven departments, the University of L'Aquila offers its over 19,000 enrolled students 69 degree courses (divided between first and second level degrees), nine research doctorate programs, specialization schools, specializing master courses, and vocational courses. The faculty includes about 600 professors and researchers.

On April 6, 2009, the devastating earthquake that hit L'Aquila brought death and destruction to the University of L'Aquila, with 55 students killed (13, 14). Part of the University staff restarted their activities 3 days after the earthquake, but the process of reconstruction of some damaged University buildings is still going on.

Since March 16, due to the difficulty of conducting face-to-face interventions during lockdown, the service was provided via a digital platform to students and young people (https://www.univaq.it/section.php?id=530; http://sacsunivaq.altervista.org/index.html).

The project proposed a free online emotional support service and was promoted through various channels (e-mail, WhatsApp, Facebook, and university institutional site).

Students seeking help could send an e-mail to the SACS and register in the protected digital space “#IoRestoaCasa” (“#istayathome”) after receiving a personal confirmation e-mail.

In the first step, the students were required to fill a short form about the main socio-demographic and clinical information, including age, gender, place of residence, off-site student condition (students were attending university in a different location, often very distant, from their residence) at the time of the lockdown, and previous mental health services contact, including prescription of psychopharmacological treatment. Furthermore, the students were asked to complete an assessment battery.

The second step included a narrative diary. The students were asked to write down the difficulties they were experiencing by responding to the following narrative stimuli, adapted from the narrative-based medicine questions and prompts (15, 16):

1. What are your main worries?
2. How is this situation affecting your life?
3. What kinds of unpleasant emotions are you feeling?
4. What kinds of unpleasant thoughts go through your mind?
5. How can we help you?

Once the responses were filled in, the person had a clinical virtual “room” with the professionals and through a protected messaging and video-chat system to communicate, according to a shared calendar. Students could use their own digital diary whenever they wanted it.

The study included 103 students consecutively referred in the almost 2-month period of the Italian lockdown (from March 16, 2020 to May 4, 2020).

All the 103 students included in the study entered the platform, filled their socio-demographic and clinical form, completed the assessment battery (first step), and answered the narrative stimuli entering their virtual room with the therapist (second step). The video sessions included counseling, problem solving, stress management strategies, and lifestyle suggestions. For each student, a weekly session lasting 60 min was planned.

After the considered period (March 16–May 4, 2020), eight (7.7%) of them did not enter the third “step” of the intervention, that is, the proposed CBT intervention for anxiety and/or the weekly planned video consultations with professionals. These students dropped the third step, not considered in our current paper, and preferred to have only the professional's video consultations.

If students showed high levels of anxiety or depression, they were invited to access the structured online CBT intervention for anxiety or to plan video sessions with the professionals.

### Assessment Battery

The following measures were administered to all participants at the entry in the platform.

#### Traumatic Distress

The Impact of Event Scale-Revised (IES-R) is one of the most widely used self-report measures in the field of traumatic stress (17). The IES-R consists of 22 items with a 5-point Likert-type scale ranging from 0 (not at all) to 4 (often). Three subscale scores can be obtained by summing the relevant item scores: intrusion, avoidance, and hyperarousal. The total IES-R score was divided into 0–23 (normal), 24–32 (mild psychological impact), 33–36 (moderate psychological impact), and >37 (severe psychological impact) (17).

#### Anxiety and Depressive Symptomatology

The 12-item General Health Questionnaire (GHQ-12) (18–20) is the most extensively used screening instrument for common mental disorders, in addition to being a more general measure of psychiatric well-being. The GHQ-12 consists of 12 items, each one assessing the severity of a mental problem over the past few weeks using a 4-point Likert-type scale (from 0 to 3). The score was used to generate a total score ranging from 0 to 36. High scores indicated poor health. The scores fell into three categories: 0–14 = normal range, 15–19 = moderate psychological distress, and 20–36 = severe psychological distress. Graetz (21) proposed a GHQ-12 three-dimensional model that included three factors: anxiety and depression (including items 2, 5, 6, and 9), social dysfunction (including items 1, 3, 4, 7, 8, and 12), and loss of confidence (including items 10 and 11).

The Self-Rating Anxiety Scale (SAS) (22) comprises 20 items that investigates anxiety symptomatology, including five items that investigate well-being (the latter require reversed scores). The items are evaluated on a 4-point Likert scale (ranging from 1 = “nothing or only for a short time” to 4 = “continuously or most of the time”). The total raw scores range from 20 to 80. Higher scores are associated with greater severity of symptoms. The clinical interpretation of the level of anxiety is as follows: 20–44 = normal range, 45–59 = mild to moderate anxiety level, 60–74 = marked to severe anxiety level, and 75–80 = extreme anxiety level.

The Beck Depression Inventory-II (BDI-II) (23) is a 21-item inventory that measures the severity of self-reported depression over the prior 2 weeks; its item content corresponds to criteria for the diagnosis of depressive disorders as specified in the Diagnostic and Statistical Manual of Mental Disorders IV, DSM-IV. Items are structured on a 4-point scale, ranging from zero points (symptom not present) to three points (symptom strongly present). Thus, a BDI-II total score from 0 to 13 points represents normal to minimal depression, from 14 to 19 points indicate mild depression, from 20 to 28 points indicate moderate depression, and from 29 to 63 points indicate severe depression.

A Concentration Impairment Index, CII, was calculated using the sum of item 1 of the GHQ-12 and item 19 of the BDI-II (range 0–6). At the beginning of the study, the evaluation of attention and concentration abilities were not considered. Based on the students' recurring reports during the clinical consultations, we found it useful to deepen these data by referring to the available quantitative measures, thus calculating a Concentration Impairment Index.

### Internet-Guided Intervention *via* a Protected Digital Platform

The platform PSYDIT.COM is a protected digital environment that brings together all the tools necessary for psychotherapy, ensuring total confidentiality of the health data as also provided by the European General Data Protection Regulation n. 2016/679. The PSYDIT.COM platform is an IT-telematic system that allows professionals and users to follow a treatment in the context of clinical practice.

PSYDIT.COM enhances the ease of digital communication, transferring it from a random, unprotected, and unmanaged context, such as emails or WhatsApp, to a communication and listening path structured and protected from the point of view of privacy.

Our intervention was administered via the PSYDIT.COM platform involving combined modalities of online therapy (synchronous and asynchronous, automatic and interpersonal, narrative, and cognitive behavioral strategies suggestions). The platform also included the following: (1) digital narrative diary available to the user to tell his/her story, through a guided tour of narrative stimuli about cognitive, emotional, and behavioral states; (2) messaging and video-counseling sessions, based on a shared calendar; and (3) a structured cognitive behavioral therapy program for anxiety (CBT). In this study, we focused on narrative dimensions analyses, using the first two functions of the platform, and on psychological distress.

The platform allows the professionals of the research team, with the involvement of all of them, to have access to the user's history and data, use a system of shared notes not visible to the users, and have a video chat for discussion or for teleconsultation.

Messages, video chats, and diary are included in an environment designed for interaction and aimed at clear and shared clinical and care objectives, which not only protect the professionals but also the user. The professionals were committed to answer within 24 h (except for weekends). The narration was used for personalization of the diagnostic-therapeutic path and was part of the user journey, in the most suitable phases and for which it is more important to enhance the user's narration. Due to its nature, such an intervention cannot be used in health emergency situations. The users were informed that they could not use the PSYDIT.COM platform to report situations of malaise or a condition that required rapid help. In these cases, the users were required to use the usual first aid and emergency medical channels.

### Data Analysis

#### Quantitative Data

Parametric and non-parametric statistics were utilized in data analysis. Chi-square test and one-way analysis of variance (ANOVA) were conducted to examine the differences in socio-demographic variables and psychopathological variables, as measured by the IES-R, GHQ-12, SAS, BDI-II, and CII, based on gender differences. Spearman correlation was performed to measure the strength and direction of the association between standardized quantitative measures (scores of GHQ, BDI-II, SAS, IES-R, and CII), qualitative variables (emotion/feeling and thinking styles), as assessed though the digital narrative diaries, and the duration of the COVID-19 home confinement.

Regression analyses were conducted for identifying potential predictors of the traumatic impact of COVID-19 lockdown. Logistic regression was used to test one predictive model. We included three blocks of variables. In step 1, socio-demographic and clinical data (women gender, age group, father and mother years of education, previous contacts with mental health services (MHS), and taking an antidepressant treatment) were included as potential predictors. Age was coded into two categories (19–21 years and 22 years and above). This categorization was based on the assumption that women and younger people might be more at risk for developing traumatic consequences. Education of relatives, indirect indicator of socio-economic status, was coded into two categories (8 years or less and more than 8 years of education). Previous contact with MHS and taking an antidepressant treatment were coded into two categories (no/yes). In step 2, distressful lockdown conditions, such as having been “locked” far from the family, were coded into two categories (no/yes), and months of the home confinement (coded into two categories first month and second month) were included as potential predictors. In step 3, we included data related to the subjects' personal cognitive thinking styles coded into two categories (yes/no).

We conducted odds ratios with 95% confidence intervals for the logistic regression analysis. Statistical analyses were performed using SPSS 26.0 (SPSS Inc., Chicago, IL, USA).

#### Qualitative Data

Narrative data analysis of qualitative components of the study was performed to identify, through significant keywords and phrases, psychological and psychosocial contents (stressful events, common affective, and cognitive patterns) experienced during the COVID-19 lockdown, reported on the digital diary of each student. In the research team meetings, the clinical psychologists (LG, SM, and DB) read and re-read several times the digital diaries. They identified and organized themes into emotional and cognitive thinking style pre-defined clusters according to the cognitive behavioral paradigm, which describes how people's perceptions of or spontaneous thoughts about situations influence their emotional, behavioral (and often physiological) reactions (24, 25). Findings were then compared and discussed by the team until consensus on coding was reached. For each student, a scoring sheet was filled in with related examples (0 = absent; 1 = present).

## Results

### Participants

The main socio-demographical, living, and clinical conditions of our University student sample are reported in Table 1.

**Table 1:** Description of the main socio-demographical, living, and clinical conditions of our university students sample.

**Variables**	
**Sex**, ***n*** **(%)**	
Women	84 (81.6)
Males	19 (18.4)
**Age (SD)**	22.5 (3.33)
**Relatives' education, years (%)**	
**Father**	
<8 years	29 (28.2)
>8 years	62 (60.1)
Missing	12 (11.7)
**Mother**	
<8 years	22 (21.3)
>8 years	69 (67.0)
Missing	12 (11.7)
**University degree courses**, ***n*** **(%)**	
Health professions	78 (75.6)
Medical school	5 (4.9)
Economics	5 (4.9)
Humanistic courses	5 (4.9)
Scientific courses	4 (3.9)
Psychological courses	3 (2.9)
Engineering courses	3 (2.9)
**Living situation**, ***n*** **(%)**	
Living with family	21 (20.4)
Off-site students (students attending university in a different location, often very distant, from their residence)	82 (79.6)
Off-site students “blocked” far from families during the lockdown period	18 (17.5)
**Previous psychological and psychiatric contacts with mental health services**, ***n*** **(%)**	23 (22.3)
**Students taking antidepressant treatments**, ***n*** **(%)**	8 (7.8)

More than 80% of the sample included women, statistically younger than male students (22.06 SD 3.11 vs. 24.37 SD 3.67; *F* = 6.952; *p* = 0.006).

In this study, almost 80% were off-site students, and in this subgroup, 18 (22%, 14 women and 4 men) were “blocked” in L'Aquila, far from their families for the entire duration of the lockdown.

More than three-quarter students were enrolled in the health professions degree courses. More than 20% of students had previous psychological and psychiatric contacts with mental health services (MHS), and around 8% of them were taking a psychopharmacological antidepressant treatment.

### Impact of Event Scale-Revised

Statistics related to the Impact of Event Scale-Revised (IES-R) are presented in Table 2. Around 20% of students experienced this lockdown as a traumatic experience. The more distressing symptoms (mean scores higher than 1.5) reported were hyperarousal (13.6%), intrusion (13.6%), and avoidance (9.7%).

**Table 2:** Statistics of traumatic, anxiety, and depressive symptomatology measures, assessed through the Impact of Event Scale-Revised 22 items (IES-R2), 12-item general health questionnaire (GHQ-12), self-rating anxiety scale (SAS), and beck depression inventory-II (BDI-II).

**Measures**	**Total sample**	**Men**	**Women**	
Impact of Event Scale-Revised (IES-R), total score mean (SD)	13.5 (13.2)	8.8 (7.69)	14.5 (13.9)	*F* = 2.973; *p* = 0.088
IES-R score 0–23, normal profile (%)	81 (78.6)	18 (94.7)	63 (75)	
IES-R score 24–32, mild psychological impact (%)	8 (7.8)	1 (12.5)	7 (8.3)	
IES-R score 33–36, moderate psychological impact (%)	4 (3.9)	0	4 (4.8)	
IES-R score >37, severe psychological impact (%)	10 (9.7)	0	10 (11.9)	chi-square = 4.122; *p* = 0.249
12-item General Health Questionnaire (GHQ-12), total score mean (SD)	14.33 (6.58)	12.2 (6.4)	14.8 (6.5)	*F* = 2.325; *p* = 0.130
GHQ-12 score 0–14, normal profile (%)	66 (64.1)	14 (73.6)	52 (61.9)	
GHQ-12 score 15–19, moderate distress (%)	13 (12.6)	1 (5.3)	12 (14.3)	
GHQ-12 score 20–26, severe distress (%)	24 (23.3)	4 (21.1)	20 (23.8)	chi-square = 1.386; *p* = 0.500
The Self-Rating Anxiety Scale, (SAS) total score mean (SD)	42.7 (9.8)	40.6 (8.5)	43.2 (10.1)	*F* = 1.053; *p* = 0.307
SAS score 0–44, normal profile (%)	66 (64)	15 (78.9)	51 (60.7)	
SAS score 45–59, mild–moderate anxiety (%)	29 (28.2)	3 (15.8)	26 (31)	
SAS score 60–74, marked–severe anxiety (%)	8 (7.8)	1 (5.3)	7 (8.3)	chi-square = 2.257; *p* = 0.323
Beck Depression Inventory-II (BDI) total score mean (SD)	10.8 (10.9)	6.42 (5.3)	11.86 (10.9)^*^	*F* = 4.427; *p* = 0.038
§* Item 1. Sadness*	0.41 (0.66)	0.11 (0.31)	0.48 (0.70)^*^	*F* = 5.038; *p* = 0.027
*Item 5. Feelings of guilt*	0.55 (0.77)	0.21 (0.41)	0.63 (0.81)^*^	*F* = 4.771; *p* = 0.032
*Item 7. Self-dislike*	0.39 (0.74)	0.05 (0.29)	0.46 (0.79)^*^	*F* = 4.926; *p* = 0.029
*Item 11. Agitation*	0.62 (0.65)	0.05 (0.29)	0.69 (0.65)^*^	*F* = 5.224; *p* = 0.022
*Item 20. Tiredness*	0.50 (0.64)	0.21 (0.41)	0.56 (0.66)^*^	*F* = 4.782; *p* = 0.031
BDI-II score 0–13, absence of depressive symptoms (%)	76 (73.8)	17 (89.5)	59 (70.3)	
BDI-II score 14–19, mild depression (%)	10 (9.7)	2 (10.5)	8 (9.5)	
BDI-II score 20–29, moderate depression (%)	9 (8.7)	0	9 (10.7)	
BDI-II score >30 severe depression (%)	8 (7.8)	0	8 (9.5)	chi-square = 4.683; *p* = 0.200

§ *Reported only statistically significant items*.

* *p < 0.05*.

IES-R scores of female students were higher than those of the male students but did not reach a statistically significant difference by gender. We found a statistically significant difference between the students with previous psychological/psychiatric contacts reporting higher IES-R scores compared to students at their first contact with our service (ANOVA: 19.08 SD 15.97 vs. 11.91 SD 11.93; *F* = 5.506; *p* = 0.021). We found no statistically significant difference between the off-site students blocked in L'Aquila and the students who lived/returned to their family.

### The 12-Item General Health Questionnaire

Table 2 summarizes the results of the GHQ-12 scores. No statistically significant difference was found between gender and lockdown housing placement, respectively. In the student subgroup scoring higher than 14, based on the model of Graetz, the highest scoring dimension was anxiety and depression (mean score 1.98, SD = 0.47), followed by social dysfunction (mean score 1.77, SD = 0.45) and loss of confidence (mean score 0.50, SD = 0.29). We found a statistically significant difference between the students with previous psychological/psychiatric contacts reporting higher GH-12 total scores compared to students at their first contact with our service (ANOVA: 17.65 SD 6.32 vs. 13.38 SD 6.37; *F* = 8.059; *p* = 0.005).

### The Self-Rating Anxiety Scale

The mean overall SAS of the sample was 42.7 (SD = 9.8). Furthermore, 36% of the student sample reported a SAS score equal to or higher than 45, without a statistically significant difference by gender and lockdown housing placement (Table 2). We found no statistically significant difference between the students with previous psychological/psychiatric contacts and the students at their first contact with our service.

### Beck Depression Inventory II

The BDI-II showed three main areas impaired in our sample: changes in sleeping pattern (reported by 68% of the total sample), lack of concentration (67%), and loss of energy (58.6%).

Further, almost 30% of students reported the presence of depressive symptomatology (Table 2).

A statistically significant difference was found in regard to gender; female students showed higher scores compared to those of male students, with respect to the total score of BDI-II, sadness, feelings of guilt, self-dislike, agitation, and tiredness. Students with previous psychological/psychiatric contacts showed higher BDI-II total scores compared to the students at their first contact with our service (ANOVA: 15.22 SD 10.28 vs. 9.61 SD 10.09; *F* = 5.464; *p* = 0.021).

We found no statistically significant difference between the off-site students blocked in L'Aquila and the students who lived/returned to their family.

### The Concentration Impairment Index

The Concentration Impairment Index showed a mean score of 2.56 DS = 1.49. No statistically significant difference in gender and lockdown housing placement was found. We found a statistically significant difference between the students with previous psychological/psychiatric contacts scores complaining a worse functioning and reporting a higher CCI score compared to students at their first contact with our service (ANOVA: 3.26 SD 1.32 vs. 2.36 SD 1.48; *F* = 6.839; *p* = 0.010).

### Qualitative Analysis

Narrative data analysis of qualitative components of the study identified, through significant key words and phrases, common affective and cognitive patterns experienced after a traumatic event. Three main psychosocial areas emerged from narrative diaries:

- Stressful factors affecting student's mood (adjustment to the new academic activities, conflicts with family members, and lack of autonomy related the COVID-19 lockdown);
- The emotions/feelings experienced by students during lockdown (fear/anxiety, sadness, anger, boredom, guilt, loneliness, and happiness);
- The cognitive responses shown by students in the evaluation of the health emergency and related factors (thinking styles as “All-or-nothing—Global negative evaluations of themselves or others,” “Catastrophizing—overestimation of risk,” “Intolerance of uncertainty,” and “Structured Positive Style”).

The main stressful factors reported by students were the following: adjustment to the new academic activities (24, 23.3%), lack of autonomy relating the COVID-19 lockdown (20, 19.4%), and conflicts with family members (7, 6.8%).

The emotions/feelings and the cognitive styles that emerged from the participants' digital diaries about outbreak experiences, according to our model, were registered and analyzed.

The emotion identified from the digital diaries were sadness, fear/anxiety, anger, guilt, boredom, loneliness, and happiness.

Table 3 shows the expressed emotions/feelings in the digital diaries, accurately identified by the therapists, their distribution percentage, and some written examples. The three main emotions/feelings more frequently reported were sadness, fear/anxiety, and loneliness.

**Table 3:** Emotions/feelings reported by students in their digital narrative diaries during the lockdown.

**Emotions/feelings**	**Student sample (*N* = 103)**	**Some examples from narrative diaries**
Sadness	63 (61.2%)	“*Sometimes I struggle to get out of bed. I don't even want to play video games, to sit down to watch a movie or TV series, also if it seems interesting and I say that I would like to do it.”* (User 2)“*The anguish and sadness assail me when I think I am about to finish the exams, and I do not have a degree thesis. At present, because of this emergency, I don't know if I will be able to carry out my internship. I don't know if I will be able to graduate or if I will remain enrolled in university because I am unable to do anything except study*.” (User 62)
Fear/anxiety	54 (52.4%)	“*I am currently anxious and worried about my mother as she works in the hospital and is in contact with potentially infected people every day*.” (User 7)“*The situation is impacting the impossibility of using hospital services. I am apprehensive and anxious about my health, since my medical visits, which I had to undergo, have been postponed to a later date. Furthermore, I am currently distant from my family who do not have easy access to the internet, thus being able to interact very little and rarely with them*.” (User 47)
Loneliness	32 (31.1%)	“*I am afraid of being totally alone, of being excluded, replaced and unable to maintain solid ties with the significant persons around me. Some days I really think that I am destined to remain alone and to have only circumstantial people around, without ever creating deep and lasting relationships.”* User (User 46).“*I feel really alone! I really miss my sister and my boyfriend who are away because of work! In 10 years of relationship, it is the first time that he happens to stay away for so long. !!!” (User 6)*
Anger	18 (17.5%)	“*These days, I am tormented by the ghosts of the past, I think back on my failures and I am taken by growing anger.” (User 3)“How can I not think negative? Not to be pessimistic? Can you explain it to me? But if every time I am fine then something negative must happen., forget it. I can't take it anymore. what the f…………… !!!.” (User 100)*
Boredom	13 (12.6%)	“*Living days as if they were all the same starts to get tired and being away from home and my family is more difficult than usual.”* (User 72)“*Some days are heavier than others and I can't do anything other than stay on the bed and think about filling myself with questions that will never be answered*.” (User 46)
Guilty	8 (7.8%)	“*Following the health emergency, I decided to return home by ending the Erasmus experience that I did so much to achieve. I feel guilty because I have not been brave, making a decision that will have significant consequences for my academic future.”* (User 52)“*I feel guilty because I know I could be a better person, but I can't forget this sadness, I feel selfish because I wish I could help instead of feeling I need help in this situation.”*Stressful factors -family problems (User 99)
Happiness	5 (4.9%)	“*I have radically changed my daily habits; I had to reorganize my days trying to make them as productive as possible. I am happy because I am learning to know myself and my family better, since we had never spent so much time together.” (User 60)“I'm an offsite student and I haven't been home since January. Now, I'm at home and I'm happy to be able to spend some time with my family, but I'm sorry I can't go out and see all my friends again.”* (User 28)

Table 4 shows the main thinking styles (as described below) of our student sample, their distribution percentage, and some participants' verbatim accounts.

**Table 4:** Thinking styles reported by students in their lockdown digital narrative diaries.

**Thinking styles**	**Student sample (*N* = 103)**	**Some examples from narrative diaries**
Intolerance of uncertainty	47 (45.6 %)	“*The frequent unpleasant thought is that of not being able to achieve my goals, because I have the feeling that my life is in stand-by right now, it does not go forward or backward.”* (User 88)“*I am worried about uncertainty, having to always be careful and fear even when going out respecting the rules that something can happen or become infected. I am also worried that according to experts the “peak” has still arrived and therefore the situation has yet to get worse.”* (User 54)
Optimistic style	27 (26.2%)	“*This situation is allowing me to spend a lot of time on myself and on my well-being, something that I haven't been able to do for a long time”*. (User 88)“*I try to get strength every day with my family, helping each other and not letting ourselves be discouraged. There is a bit of concern, but for now nothing that I can't manage easily. I think we need to be confident, and everything will be fine.”* (User 15)
All or nothing/devaluation of self or others	25 (24.3%)	“*In my days, there is no margin of error although apparently I may seem relaxed and available for recreational activities (walks, sports, lunch with my girlfriend), whenever a break lasts too long or that I wake up late in the morning or that I take the phone for whatever reason the whole day in my mind has been lost and there is no possibility of correction, it is all lost now*.” (User 62)“*In this difficult situation, I am not doing anything to help people! I'm a useless person!!!”* (User 13)
Catastrophizing/overestimation of risk	10 (9.7 %)	“*All the sacrifices I made to be a better person, the person I wanted to be, all the good intentions and progress that I made in the last year, after years of dissatisfaction and sadness, vanished. Now there is nothing left, I feel failed and oppressed. I don't know if I will ever be able to get my life back in hand as I was able to do after so many efforts. Chest pain will probably not stop with the end of the quarantine.”* (User 52)“*I am concerned about not returning to a “normal” situation, the fact that they do not give us back our freedoms, it makes me feel bad not to be able to do this or that. I have a feeling that this situation will never change and that torments me.”* (User 89)“*I am currently concerned about the situation that the whole nation is experiencing. In particular, I am afraid that some family member or loved one (myself included) could be infected without the possibility of being treated and in the worst case, of dying in total solitude. Often, I happen to imagine the consequences that may occur in the future, especially in a situation in which even the basic needs will start to fail and we will be reduced to living in conditions of pure subsistence. Or sometimes I think that if they were to reopen every national structure and everything were to return to normal, there could be contagions and relapses again especially for us students who will have to attend public and crowded places like the university.”* (User 26)

#### All-or-Nothing/Global Negative Evaluations of Themselves

This distortion (also known as “black-and-white thinking”) manifests as an inability or unwillingness to see shades of gray. In other words, you see things in terms of extremes—something is either fantastic or awful; you believe you are either perfect or a total failure.

#### Catastrophizing/Overestimation of Risk

This occurs when the person thinks about worst-case scenarios as if they are likely-case scenarios, and they self-induce a great deal of distress over anticipated hardships and losses that may be unlikely.

#### Intolerance of Uncertainty

Intolerance of uncertainty is defined as the cognitive style related to the dispositional fear underlying emotional difficulties and resulting in anxiety in cases where the unknown is perceived intensely (26).

#### Optimistic Style

Optimism is a cognitive attitude reflecting a belief or hope that the outcome of some specific endeavor, or outcomes in general, will be positive, favorable, and desirable.

### Correlation Between Quantitative and Qualitative Variables

Table 5 shows the correlations of all investigated quantitative, emotional, and cognitive variables and the duration of home confinement.

**Table 5:** Correlation analyses and qualitative and quantitative measures.

	**GHQ total** ** score**	**BDI-II Total** ** score**	**BDII-livelli di** ** gravità**	**SAS total score**	**IES-R total score**	**IES-R avoidance**	**IES-R intrusion**	**IES-R** ** hyperarousal**
All_or_nothing	0.404**	0.381**	0.423**	0.341**	0.231*	0.1	0.143	0.350**
*P*-value	<0.001	<0.001	<0.001	<0.001	0.019	0.313	0.149	<0.001
Catastrophic_thought	0.184	0.213*	0.280**	0.144	0.098	0.07	0.049	0.143
*P*-value	0.069	0.031	0.004	0.148	0.324	0.481	0.624	0.15
Intolerance_uncertainty	0.067	0.066	−0.007	0.069	0.147	0.157	0.144	0.071
*P*-value	0.501	0.508	0.942	0.487	0.139	0.113	0.147	0.475
Optimistic_style	−0.338**	−0.256**	−0.259**	−0.248*	−0.051	0.081	0.083	−0.233**
*P*-value	<0.001	0.009	0.008	0.011	0.609	0.418	0.405	0.018
Sadness	0.024	0.048	0.114	−0.035	−0.016	−0.021	−0.045	0.024
*P*-value	0.811	0.63	0.251	0.724	0.869	0.831	0.65	0.813
Fear_anxiety	0.202*	0.145	0.118	0.151	−0.054	−0.094	−0.111	0.07
*P*-value	0.041	0.144	0.235	0.128	0.588	0.343	0.266	0.483
Anger	0.228*	0.231*	0.126	0.167	0.127	0.062	0.05	0.168
*P*-value	0.021	0.019	0.205	0.092	0.2	0.532	0.613	0.089
Happiness	−0.133	−0.16	−0.133	−0.196*	−0.045	0.065	0.121	−0.174
*P*-value	0.18	0.107	0.181	0.047	0.653	0.512	0.222	0.079
Guilty	0.135	0.158	0.158	0.174	0.012	−0.114	−0.061	0.063
*P*-value	0.174	0.11	0.111	0.079	0.907	0.252	0.538	0.526
Boredom	−0.075	−0.028	0.020	−0.123	−0.125	−0.148	−0.182	−0.018
*P*-value	0.452	0.782	0.843	0.215	0.208	0.136	0.066	0.859
Loneliness	0.176	0.053	0.096	−0.044	−0.011	−0.047	−0.04	0.038
*P*-value	0.075	0.595	0.333	0.66	0.91	0.64	0.687	0.706
Quarantine_day	0.204*	0.182	0.213*	0.147	0.340**	0.330**	0.298**	0.306**
*P*-value	0.039	0.066	0.031	0.139	<0.001	0.001	0.002	0.002
Concentration impairment index	0.685**	0.715**	0.624**	0.527**	0.420**	0.07	0.105	0.737**
*P*-value	<0.001	<0.001	<0.001	<0.001	<0.001	0.482	0.29	<0.001

The table reports correlation coefficients (Spearman's ρ) and statistical significance (*p < 0.05; **p < 0.01). GHQ, General Health Questionnaire; BDI-II, Beck Depression Inventory-II; SAS, Self-Rating Anxiety Scale; IES-R, Impact of Event Scale-Revised.

Positive and statistically significant correlations of the problematic thinking style “all or nothing” were found between psychological distress, measured by the GHQ total score; anxiety symptoms, as measured by the SAS total score; and post-traumatic symptoms, measured by the IES total score. Hence, it was concluded that this negative cognitive pattern could promote negative emotional responses. Correlations analyses showed positive and significant correlations of “all or nothing” and “catastrophism” thinking styles with depressive symptoms and severity, as measured by BDI total scores and their severity levels, suggesting that both these cognitive styles could contribute to the maintenance or reinforcement of low mood.

Furthermore, a high level of psychological distress (GHQ total score) correlated positively and significantly with fear/anxiety and anger, showing that these negative feelings contributed to a condition of daily suffering. Anger also correlated with depression symptoms, as measured by the BDI total score.

Negative and statistically significant correlation of the optimistic thinking style with psychological distress, anxiety symptoms, depressive symptoms and their relative severity levels, and with the post-traumatic dimension of hyperarousal was found. A negative and statistically significant correlation between the positive feeling “happiness” and anxiety symptoms was found.

If any statistically significant difference was found in thinking styles by gender, a higher statistically significant proportion of off-site students (55.6%) blocked in L'Aquila expressed feelings of loneliness in their digital diaries compared to the students who spent the lockdown with their family (25.9%) (chi-square = 6.107; DF = 1; *p* = 0.013).

Concentration impairment, as measured using CII, positively and significantly correlated with psychological distress, anxiety symptoms, depressive symptoms, and post-traumatic symptoms, especially in the hyperarousal dimension, depicting relevant impact of psychopathology on cognitive functioning. No statistically significant difference between gender and lockdown housing placement was found. Students showing an optimistic style showed statistically significant lower concentration impairment scores compared to their colleagues (ANOVA: 1.88 SD = 1.25 vs. 2.80 SD = 1.50; *F* = 7.973, *p* = 0.006).

Based on the correlation analyses, the level of psychological distress (GHQ total score), depressive and severity symptoms (BDI total and its severity level), and the post-traumatic symptomatology tended to increase with the progression of the days of lockdown (home quarantine).

### Predictors of Traumatic Symptomatology

The predictive model shown in Table 6 is the result of the logistic regression analysis for predicting the traumatic impact of COVID-19 lockdown from the IES-R scale (total score > 23).

**Table 6:** Logistic regression analysis for predicting the traumatic impact of COVID-19 lockdown from the IES-R scale (total score >23).

	**Step 1**					**Step 2**					**Step 3**				
	***B***	***p***	**Exp(B)**	**CI 95% *L***	**CI 95% *U***	***B***	***p***	**Exp(B)**	**CI 95% *L***	**CI 95% *U***	***B***	***p***	**Exp(B)**	**CI 95% *L***	**CI 95% *U***
**Sex**															
Men/women	1.796	0.101	6.028	0.703	51.677	2.095	0.061	8.127	0.906	72.9	1.975	0.137	7.21	0.535	97.177
**Age** **≤21 years**															
No/yes	0.013	0.879	1.013	0.86	1.193	0.01	0.917	1.01	0.843	1.209	0.059	0.556	1.06	0.872	1.289
**Father's years of education**															
<8 years/>8 years	−0.011	0.987	0.989	0.268	3.654	0.11	0.874	1.117	0.285	4.375	0.262	0.741	1.3	0.275	6.152
**Mother's years of education**															
<8 years/>8 years	−0.704	0.364	0.495	0.108	2.259	−1.217	0.147	0.296	0.057	1.536	−1.268	0.157	0.282	0.049	1.627
**Previous contact with MHS**															
No/yes	0.398	0.23	1.489	0.778	2.851	0.346	0.323	1.414	0.711	2.813	0.644	0.08	1.904	0.926	3.912
**Taking antidepressant treatment**															
No/yes	−1.006	0.451	0.366	0.027	4.992	−1.058	0.442	0.347	0.023	5.148	−2.535	0.117	0.079	0.003	1.884
**Locked student**															
No/yes						0.42	0.565	1.521	0.364	6.359	0.894	0.271	2.445	0.498	12.017
**Quarantine month**															
First month/second month						1.313	**0.025**	**3.716**	1.179	11.705	0.888	0.177	2.429	0.669	8.82
**All-or-nothing thinking style**															
No/yes											1.704	**0.03**	**5.495**	1.179	25.598
**Catastrophic thinking style**															
No/yes											1.097	0.155	2.995	0.66	13.596
**Intolerance-uncertainty**															
No/yes											0.44	0.507	1.552	0.423	5.694
**Optimistic style**															
No/yes											1.55	0.049	4.713	1.008	22.048

*In bold significant values are reported*.

Within the first step, among the socio-demographical, living, and clinical variables, none of the variables entered in the model showing a statistically significant predictive power. In step 2, including variables related to the trauma “exposition,” the likelihood of a positive estimate of traumatic symptomatology increased around four times during the second month of home confinement (Table 6). The duration of the confinement seemed to have significant predictive power in our model since we did not enter the personal cognitive thinking styles. In the third step, only the “all-or-nothing” cognitive style showed a significant predictive power, and the likelihood of a positive estimate of traumatic reaction increased to more than five times.

The values of Nagelkerke's *r*^2^ for the three blocks within the model in Table 6 are 0.12 for step 1, 0.206 for step 2, and 0.335 for step 3.

## Discussion

To the best of our knowledge, this study is a first in investigating quantitative emotional and cognitive aspects and qualitative psychopathological data on a digital platform during the lockdown following the Italian outbreak of COVID-19. Until now, narrative medicine worked on interviews, written reports, and storytelling. Also, the study contributed to the identification of potential predictors of post-traumatic distress in a sample of university students seeking help to the counseling and consultation service.

First, we analyzed the emotional and cognitive experiences and the psychopathological symptomatology of youths during the occurrence of the Italian lockdown due to COVID-19. A little more than 20% of our students experienced this lockdown as a traumatic experience. Furthermore, 36% of the students reported to be psychologically distressed and suffering from anxiety symptoms, whereas 26% showed depressive symptomatology.

In this study, more than 80% of the female students were more likely to ask for help, but we did not report a higher proportion of female students affected. About one-fifth of the students that had previous psychological and psychiatric contacts with MHS showed a more severe traumatic and depressive symptomatology. The three main areas impaired were changes in the sleeping pattern (68%), lack of concentration (67%), and loss of energy (58.6%).

Our study confirms the effect of COVID-19 on young people, showing a high, similar proportion of youth suffering from psychological problems, nearly 40%, as seen in a Chinese study (41.4%) (3), and a higher proportion suffering from post-traumatic symptoms as compared to the Chinese population (14.4%). In the scientific literature, women seem more likely to show symptoms of PTSD related to traumas, and Mazza et al. (27) confirmed this data during the Italian COVID-19 outbreak; surprisingly, our study did not show such evidence. Furthermore, Liang et al. (3) found that during the COVID-19 outbreak, in their sample of youths, men scored significantly higher on psychological distress, PTSD, and negative coping scales as compared to women.

The high level of depression, anxiety, and stress symptomatology could be the basis for sleep difficulties, reported by almost 70% of our student sample, in line with the Italian survey data of 52.4% of poor sleepers registered by Cellini et al. (28). Moreover, sensation of time elongation, increased hours of information exposure, increased use of social media and websites, frequent inversion of circadian rhythms (sleeping in the morning and the afternoon), impossibility to articulate daily life in different activities, and spaces could be hypothesized to be co-responsible for sleep disturbances. Difficulties in concentration and loss of energy were most reported by a large part of our student sample in their digital diaries, with a reduced progression in studies. Psychological distress was positively and significantly correlated with concentration and attentive difficulties, showing that psychological distress could impact our ability to function correctly.

According to the correlation analyses, the level of post-traumatic, anxiety, and depressive symptoms tended to increase with the progression of the days of the lockdown period (home confinement). As if, after an initial phase of optimism, the challenges, efforts, and changes related to the event and the relative adaptation difficulties, such as difficulty in studying, family conflicts, and increased annoyance toward social restrictions, begin to emerge predominantly. Our findings confirmed the impact of the duration of quarantine on post-traumatic distress symptoms (29, 30).

The analysis of narrative digital diaries allowed to detect the “optimistic style” in around a quarter of the students of our sample (26.2%). At the time of COVID-19 pandemic, the popular expression repeated all over the world, “Everything Will Be Fine,” could be identified in such a cognitive style, inversely correlated with psychopathological distress and concentration problems.

As expected, a negative and statistically significant correlation was observed between “optimistic” thinking style and psychological distress, anxiety, depressive, and post-traumatic symptoms, showing that this positive cognition was correlated with a sense of well-being and could represent an important resilient resource for a better adjustment to stressful situations (31). According to these results, a negative and statistically significant correlation was found between anxiety and happiness, which together with the optimistic style represented an adaptive and positive response to adversity. Both optimistic style and the feeling of happiness depict the resilient strength of “Everything-Will-Be-Fine” students, who better survived the lockdown period. Feelings of loneliness were experienced by a higher proportion of off-site students “blocked” in their university town compared to the students who were able to go back home to their families.

More than 50% of our sample showed an “intolerance-of-uncertainty” style of thinking, variable recently studied during the pandemic COVID-19 outbreak (32). The authors reported the relationship of intolerance of uncertainty and mental well-being, mediated by rumination and fear of COVID-19 (32). Our data do not confirm the relationship between “intolerance of uncertainty,” which certainly has pervaded most, and the distress of our students.

The “intolerance of uncertainty” was the most represented cognitive style in our student sample, but the “all or nothing” one influenced students' well-being more negatively, directly correlated with the psychopathological distress and post-traumatic symptoms. The thinking style “all or nothing” represents a negative cognitive pattern that identifies an inability to see the alternatives in a situation or solutions to a problem and may represent an obstacle to well-being, characterizing around 25% of the sample.

Second, we investigated the variables that could predict the traumatic impact of the COVID-19 home confinement. The “all-or-nothing” thinking style was the final strongest predictor increasing by over five times the likelihood of experience post-traumatic symptomatology, confirming that maladaptive appraisals can predict severity of stress reactions after a traumatic event and mediate adaptive functioning to environmental stressors (33).

None of the socio-demographic (gender, age, and relatives' educational level) or clinical (previous contact with MHS and antidepressant treatment) variables were predictive of a potential presentation of post-traumatic symptomatology. The results of the logistic regression analysis on our selected variables also indicated that the duration of home confinement, the second month, seemed to increase of 3.7 times the risk of post-traumatic manifestations since the cognitive thinking styles were not entered in the model. The insertion of these variables modifies the “risk model” concerning such usually unexplored factors. The condition of being “locked,” home confined far from family in the university town, considered as another potential peritraumatic factor, did not enter our model.

We cannot compare our results with the results of the Chinese study of Cao et al. (5), the only study investigating predictors of the psychological impact of the COVID-19 epidemic on college students. They identified factors not investigated in our study, such as risk factors “living in urban areas,” “family income stability,” and “living with parents” and protective factors, such as “having relatives or acquaintances infected with COVID-19.”

A Spanish study investigating on university students and workers in a sample of 2,530 participants found moderate to extremely severe scores of anxiety, depression, and stress reported by 21.34%, 34.19%, and 28.14% of the respondents, respectively (34). Evaluating the psychological impact level, half of the sample obtained a score related to the psychological impact of outbreak and lockdown as moderate or severe (IES ≤ 26). The university staff presented lower scores in all measures compared to students, who have been specially impacted by the COVID-19 confinement during the first weeks of the lockdown. The authors hypothesized that students could be more concerned about their perception of the future and alarmed by their way of consuming information media, etc.

A recent Italian study investigated on psychological distress among general population during the COVID-19 pandemic and examined the potential predictive value of sociodemographic variables and personality traits. Among selected predictors of their constructed model, the student condition did not seem to represent a predictive significant variable of stress, anxiety, and depression symptoms (27), whereas significant predictors were female gender, negative affect, and detachment. Having an acquaintance infected, a history of stressful situations and medical problems, a family member infected, and young person who had to work outside their domicile presented higher levels of psychopathological symptoms.

This study has some strengths and limitations. Among the strengths, firstly, this is an early study that investigates not only psychopathological variables but also the cognitive and emotional experiences of a sample of university students. Secondly, the current study uses a protected digital platform that allowed the collection of personal experiences from “innovative” narrative diaries. Thirdly, the identification of predictors as dysfunctional cognitive styles can address targeted interventions on subgroups of a vulnerable population.

Regarding the limitations, the main limitation of this study is the sample size. The study was not presented as one out of the several Internet anonymous surveys on psychological conditions during the COVID-19 home confinement. It was addressed to students in need of help for their psychological and/or academic difficulties. Then, we can hypothesize that the access could be limited because of the need for the registration on a digital platform after sending an e-mail, the absence of anonymity, and the conduction of video consultations, implying more than a single action and an overt request of help. Moreover, the results of our study are not generalizable concerning the qualitative findings due to the difficulty of conducting a rigorous reliable qualitative narrative analysis. Although our research study on qualitative data followed the criteria for SRQR (Standard for Reporting Qualitative Research) (35), the presence of possible bias in the data analysis has to be considered, while respecting a paradigm like the cognitive behavioral one.

## Conclusion

Imposed home confinement or isolation is an unfamiliar and unpleasant experience that involves separation from friends and family and a departure from usual, everyday routines (36). Social isolation associated with home confinement can be the catalyst for many mental health sequelae, even in people who were previously well (37).

At the time of COVID-19, services are providing psychological counseling using electronic digital devices and applications (such as smartphones and chat) for help seekers, for persons affected by mental disorders, as well as their families (38), and this can represent an opportunity to improve the accessibility to psychological and mental health services, beyond the virus spreading.

Our study was based on the utilization of a digital platform that integrated quantitative and qualitative, narrative data and investigated not only psychopathological profiles in young people but also their emotional and cognitive experiences at the time of an exceptional event of forced social isolation, the COVID-19 outbreak.

If happiness and optimistic style, shown by a quarter of our students, give content to the “resilience” model, underlying the “#everythingwillbefine” message of hope, our preliminary data suggests the need of monitoring the rest of the students who showed significant difficulties instead during the COVID-19 pandemic.

The identification of the “all-or-nothing” dysfunctional cognitive style, as a robust predictor of post-traumatic symptoms, can address intervention on such a modifiable risk factor.

The implementation of psychological interventions to improve the mental health of vulnerable young subgroups during a global health emergency and to contain, as far as possible, the evolution and structuring of psychopathological profiles represents a fundamental challenge.

## Data Availability Statement

The raw data supporting the conclusions of this article will be made available by the authors, without undue reservation.

## Ethics Statement

The studies involving human participants were reviewed and approved by University of L'Aquila, Internal Review Board. The patients/participants provided their written informed consent to participate in this study.

## Author Contributions

LG, MC, and RR designed the study and wrote the protocol. AS developed and oversaw the maintenance of the digital platform. LG, DB, SM, AS, and DU followed the clinical cases and conducted clinical video-chat interviews. LG, DB, and SM analyzed the narrative data. LG and RR conducted the statistical analyses. LG, MC, and RR conducted literature research and wrote the publication. All authors contributed to the article and approved the submitted version.

## Conflict of Interest

The authors declare that the research was conducted in the absence of any commercial or financial relationships that could be construed as a potential conflict of interest.
